# Arcanobacterium pinnipediorum Strain DSM 28752 Isolated from a Harbour Seal: Complete Genome Sequence

**DOI:** 10.1128/mra.01180-22

**Published:** 2023-01-04

**Authors:** Maria Borowiak, Antonia Kreitlow, Burkhard Malorny, Mazen Alssahen, Christoph Lämmler, Ellen Prenger-Berninghoff, Christa Ewers, Ursula Siebert, Madeleine Plötz, Amir Abdulmawjood

**Affiliations:** a German Federal Institute for Risk Assessment (BfR), Department for Biological Safety, Berlin, Germany; b Institute of Food Quality and Food Safety, University of Veterinary Medicine Hannover, Hannover, Germany; c Institut für Hygiene und Infektionskrankheiten der Tiere, Justus-Liebig-Universität Gießen, Gießen, Germany; d Institute for Terrestrial and Aquatic Wildlife Research (ITAW), University of Veterinary Medicine Hannover, Hanover, Germany; University of Delaware

## Abstract

The genus *Arcanobacterium* is constantly growing as novel species are identified. In particular, harbor seals have proven to be a common reservoir for bacteria of this genus. Here, we announce the complete genome sequence of another *Arcanobacterium* species—namely, Arcanobacterium pinnipediorum strain DSM 28752, isolated from a harbor seal.

## ANNOUNCEMENT

Bacteria of the genus *Arcanobacterium* are facultatively anaerobic, asporogenous Gram-positive rods (1). Some species of this genus have been associated with human and animal disease, highlighting their pathogenic potential (2–5).

In harbor seals and other marine mammals, Arcanobacterium phocae is widespread and sometimes associated with wound infections (6, 7). Routine microbial diagnostic investigations performed as part of a program monitoring harbor seals in the German North Sea revealed that harbor seals also appear to be a natural reservoir for other *Arcanobacterium* species, as three novel species were identified—namely, Arcanobacterium phocisimile (8, 9), A. buesumense (10, 11), and A. pinnipediorum (1, 12).

Here, we present the complete genome sequence of *A. pinnipediorum* strain DSM 28752 and place it in the phylogenetic tree of the genus *Arcanobacterium*.

*A. pinnipediorum* strain DSM 28752 was isolated in 2004 from an anal swab of a living harbor seal during routine diagnostics (1, 12). The isolate was stored in the in-house culture collection of the Institute of Food Quality and Food Safety, University of Veterinary Medicine Hannover, at −80°C using the CryoInstant preservation system (VWR Chemicals, Germany). For genomic DNA extraction, the isolate was cultivated on sheep blood agar under microaerobic conditions for 48 h at 37°C. DNA was extracted from collected cell material using the MagMAX microbiome Ultra nucleic acid isolation kit (Thermo Fisher Scientific, Darmstadt, Germany). Subsequently, whole-genome sequencing was performed using Illumina and Oxford Nanopore technologies.

For all software mentioned in the following, default settings were used. For Illumina sequencing, a library was prepared using the Nextera DNA Flex kit (Illumina, San Diego, CA, USA) and sequenced in 2 × 149-bp cycles on an Illumina NextSeq 500 sequencer using the NextSeq 500/550 midoutput kit v2.5. The reads were trimmed using fastp v0.19.5, resulting in 3,118,736 high-quality reads (Q30, >91.39%).

For MinION sequencing, a library was prepared using the rapid barcoding kit (Oxford Nanopore Technologies [ONT], Oxford, UK) and sequenced on an ONT MinION device connected to ONT MinIT v19.12.5 software (including the Guppy v3.2.10 base caller) using a FLO-MIN106 R9 flow cell. The reads were trimmed using Porechop v0.2.3 (https://github.com/rrwick/Porechop) and filtered using NanoFilt v2.7.1 (13). Quality control using NanoStat v1.2.1 (13) revealed 14,032 reads with a read length *N*_50_ value of 9,798 bp and a mean read quality score of 10.8.

Both data sets were *de novo* assembled and circularized using the hybrid assembler Unicycler v0.4.8 including Pilon for polishing (14–16). The resulting genome consists of a circular chromosome with a length of 1,952,277 bp and a GC content of 50.7%. The genome sequence was annotated using the Prokaryotic Genome Annotation Pipeline v4.11 implemented in NCBI (https://www.ncbi.nlm.nih.gov/genome/annotation_prok/) (17).

**FIG 1:**
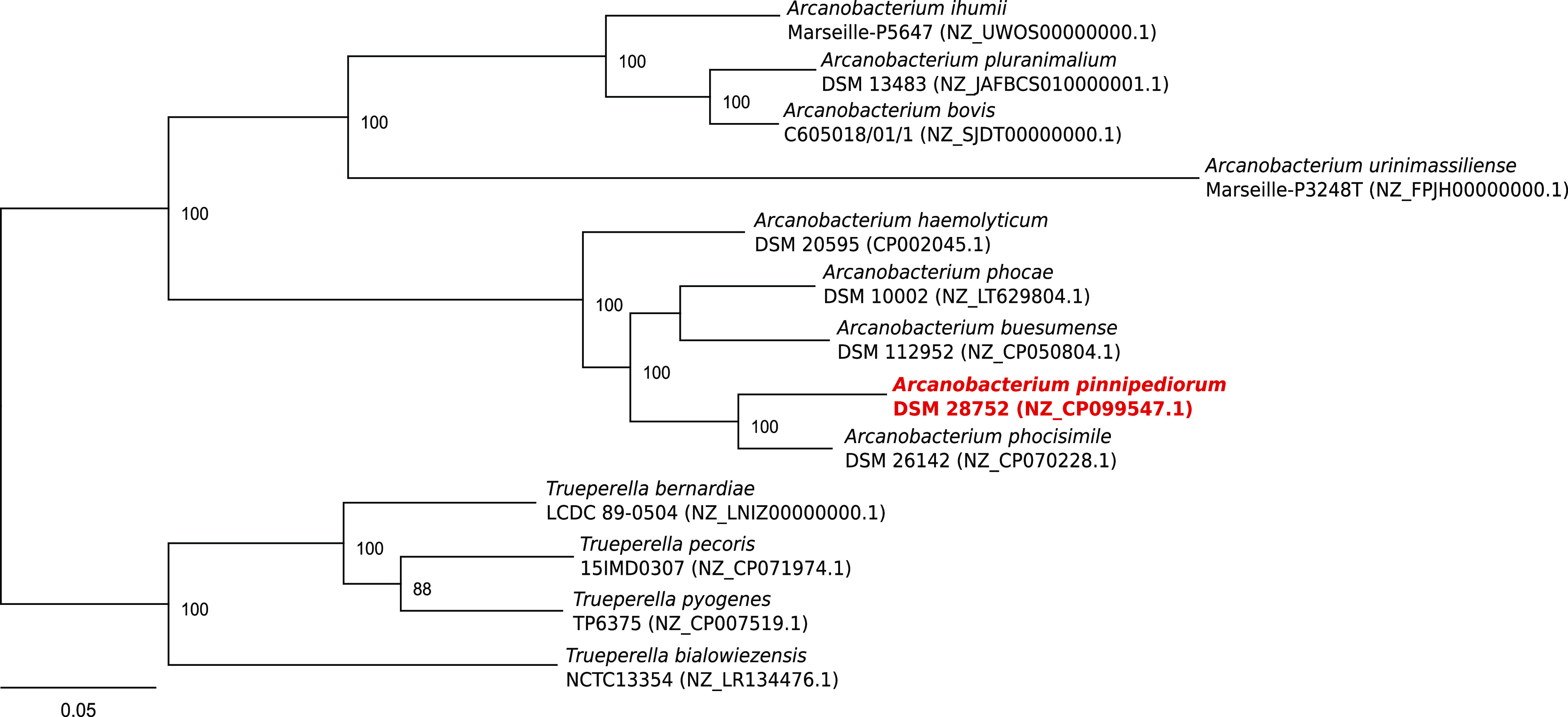
Best-scoring maximum likelihood tree based on a comparison of the amino acid sequences of 107 essential single-copy core genes of *A. pinnipediorum* strain DSM 28752, other published *Arcanobacterium* spp., and closely related *Trueperella* spp. performed using bcgTree v1.1.0. Numbers at the branches designate the bootstrap support values resulting from 100 bootstrap replicates. The tree was visualized using Geneious v2020.2.2 (Biomatters, Auckland, New Zealand), manually rooted using the *Trueperella* sp. node, and finalized using Inkscape v1.0.2.

For phylogenetic classification, the genome sequence of *A. pinnipediorum* strain DSM 28752 was compared to other *Arcanobacterium* sp. and closely related *Trueperella* sp. genomes available at NCBI. All genomes were locally annotated using Prokka v1.1.3 (18), and a maximum likelihood phylogenetic tree was constructed by amino acid sequence comparison of 107 single-copy core genes using bcgTree v1.1.0 (19) as previously described (9). The resulting phylogenetic tree (Fig. 1) revealed that *A. pinnipediorum* strain DSM 28752 is most closely related to the three *Arcanobacterium* species *A. phocisimile*, *A. buesumense*, and *A. phocae*, all originally identified in harbor seals.

### Data availability.

The complete genome sequence has been deposited at NCBI GenBank (accession number CP099547.1). The MinION and Illumina sequencing data have been deposited in the NCBI Sequence Read Archive (SRA) under the accession numbers SRX16114199 and SRX16114198, respectively.
